# A convolutional neural network-based system to classify patients using FDG PET/CT examinations

**DOI:** 10.1186/s12885-020-6694-x

**Published:** 2020-03-17

**Authors:** Keisuke Kawauchi, Sho Furuya, Kenji Hirata, Chietsugu Katoh, Osamu Manabe, Kentaro Kobayashi, Shiro Watanabe, Tohru Shiga

**Affiliations:** 1 Graduate School of Biomedical Science and Engineering, School of Medicine, Hokkaido University, N15 W7, Kita-ku, Sapporo, 0608638 Japan; 2 Department of Diagnostic Imaging, Hokkaido University Graduate School of Medicine, N15 W7, Kita-ku, Sapporo, 0608638 Japan; 3 Department of Nuclear Medicine, Hokkaido University Hospital, N15 W7, Kita-ku, Sapporo, Hokkaido 0608638 Japan; 4 Faculty of Health Sciences Biomedical Science and Engineering, Hokkaido University, N15 W7, Kita-ku, Sapporo, 0608638 Japan

**Keywords:** FDG, PET, Convolutional neural network, Deep learning

## Abstract

**Background:**

As the number of PET/CT scanners increases and FDG PET/CT becomes a common imaging modality for oncology, the demands for automated detection systems on artificial intelligence (AI) to prevent human oversight and misdiagnosis are rapidly growing. We aimed to develop a convolutional neural network (CNN)-based system that can classify whole-body FDG PET as 1) benign, 2) malignant or 3) equivocal.

**Methods:**

This retrospective study investigated 3485 sequential patients with malignant or suspected malignant disease, who underwent whole-body FDG PET/CT at our institute. All the cases were classified into the 3 categories by a nuclear medicine physician. A residual network (ResNet)-based CNN architecture was built for classifying patients into the 3 categories. In addition, we performed a region-based analysis of CNN (head-and-neck, chest, abdomen, and pelvic region).

**Results:**

There were 1280 (37%), 1450 (42%), and 755 (22%) patients classified as benign, malignant and equivocal, respectively. In the patient-based analysis, CNN predicted benign, malignant and equivocal images with 99.4, 99.4, and 87.5% accuracy, respectively. In region-based analysis, the prediction was correct with the probability of 97.3% (head-and-neck), 96.6% (chest), 92.8% (abdomen) and 99.6% (pelvic region), respectively.

**Conclusion:**

The CNN-based system reliably classified FDG PET images into 3 categories, indicating that it could be helpful for physicians as a double-checking system to prevent oversight and misdiagnosis.

## Background

FDG PET/CT is widely used to detect metabolically active lesions, especially in oncology [1, 2]. PET/CT scanners are becoming widespread because of their usefulness, whereas the number of FDG PET/CT examinations has also increased. In Japan, the number of institutes that have installed a PET/CT scanner has increased by 177 (212 to 389) from 2007 to 2017, with examinations increasing 72% from 414,300 to 711,800 [3]. In the current clinical practice, FDG PET/CT images require interpretation by specialists in nuclear medicine. As the physicians’ burden of interpreting images increases, the risk of oversight or misdiagnosis also increases. Therefore, there is a demand for an automated system that can prevent such incidents.

Image analysis using a convolutional neural network (CNN), a machine learning method, has attracted a great deal of attention as a method of artificial intelligence (AI) in the medical field [4–7]. CNN is a branch of deep neural network (so-called deep learning) techniques and is known to be feasible for image analysis because of its high performance at image recognition [8]. In a previous study using a CNN, tuberculosis was automatically detected on chest radiographs [9]. The use of a CNN also enabled brain tumor segmentation and prediction of genotype from magnetic resonance images [10]. Another study showed high diagnostic performance in the differentiation of liver masses by dynamic contrast agent-enhanced computed tomography [11]. CNN methods have also been applied to PET/CT, with successful results [12–14].

We hypothesized that introducing an automated system to detect malignant findings would prevent human oversight/misdiagnosis. In addition, the system would be useful to select patients who need urgent interpretation by radiologists. Physicians who are inexperienced in nuclear medicine would particularly benefit from such a system.

In this research, we aimed to develop a CNN-based diagnosis system that classifies whole-body FDG PET images into 3 categories: 1) benign, 2) malignant and 3) equivocal; such a system would allow physicians performing radiology-based diagnosis to double-check their opinions. In addition, we examined region-based predictions in the head and neck, chest, abdomen, and pelvis regions.

## Methods

### Subjects

This retrospective study included 3485 sequential patients (mean age ± SD, 63.9 ± 13.6 y; range, 24–95 y) who underwent whole-body FDG PET/CT (Table 1). All patients were scanned on either Scanner 1 (*N* = 2864, a Biograph 64 PET/CT scanner, Asahi-Siemens Medical Technologies Ltd., Tokyo) or Scanner 2 (*N* = 621, a GEMINI TF64 PET/CT scanner, Philips Japan, Ltd., Tokyo) at our institute between January 2016 and December 2017.

The institutional review board of Hokkaido University Hospital approved the study (#017–0365) and waived the need for written informed consent from each patient because the study was conducted retrospectively.

### Model training and testing

Experiment 1 (Whole-body): First, input images were resampled to (224, 224) pixels to match the input size of the network. After that, we trained CNN using data from the FDG PET images. CNN was trained and validated using 70% patients (*N* = 2440; 896 benign, 1015 malignant, and 529 equivocal) which were randomly selected. After the training process, the remaining 30% patients (*N* = 1045; 384 benign, 435 malignant, and 226 equivocal) were used for testing. A 5-fold cross-validation scheme was used to validate the model, followed by testing. In the model-training phase, we used “early stopping” and “dropout” to prevent overfitting. Early stopping is a method used to monitor the loss function of training and validation and to stop the learning before falling into excessive learning [15]. Early stopping and dropout have been widely adopted in various machine-learning methods [16, 17].

Experiment 2 (Region-based analysis): In this experiment, the neural network having the same architecture were trained using 4 datasets consisting of differently cropped images: (A) head and neck, B) chest, C) abdomen, and D) pelvic region, respectively. The label was malignant when the malignancy existed in the corresponding region. The label was equivocal when the equivocal uptake existed in the corresponding region. Otherwise, the label was benign. The configuration of the network was the same as in Experiment 1.

Experiment 3 (Grad-CAM [18]): We carried out additional experiments using the Grad-CAM technique, which visualizes the part activating the neural network. In other words, Grad-CAM highlights the part of the image that the neural network responds to. The same image as the original image used in Experiment 1 was used as the input image. To evaluate the results of Grad-CAM, we extracted 100 malignant patients randomly from the test cohort. Grad-CAM provided continuous value for each pixel, and we set 2 different cut-offs (70 and 90% of maximum) to contour the activated area. The Grad-CAM result was judged correct or incorrect by a nuclear medicine physician.

### Labeling

An experienced nuclear medicine physician classified all the patients into 3 categories: 1) benign, 2) malignant and 3) equivocal, based on the FDG PET maximum intensity projection (MIP) images and diagnostic reports. The criteria of classification were as follows.

1. The patient was labeled as malignant when the radiology report described any malignant uptake masses and the labeling physician confirmed that the masses were visually recognizable.
2. The patient was labeled as benign when the radiology report described no malignant uptake masses and the labeling physicians confirmed that there was no visually recognizable uptake indicating malignant tumor.
3. The patient was labeled as equivocal when the radiology report was inconclusive between malignant vs. benign and the labeling physician agreed with the radiology report. In case the labeling physician disagreed with the radiology report, the physician further investigated the electric medical record and categorized the patient into malignant, benign, or equivocal.

Finally, 1280 (37%) patients were labeled “benign”, 1450 (42%) “malignant” and 755 (22%) “equivocal”. Note that the number of the malignant label was smaller than the number of pretest diagnoses in Table 1, mainly because Table 1 includes patients who were suspected of cancer recurrence before the examination but showed no malignant findings on PET.

**Table 1:** Patient characteristics

	n (%)
Total patients	3485
Males	1954 (56.1)
Females	1531 (43.9)
Age (in years)	
Mean ± SD	63.9 ± 13.6
Range	24–95
Cancer-related biomarkers	Positive/Total (%)
AFP	16/167 (9.6)
CA19–9	177/591 (29.9)
CEA	282/889 (31.7)
CYFRA	138/402 (34.3)
NSE	381/621 (61.4)
PIVKA-II	24/135 (17.8)
Pro-GRP	95/540 (17.6)
PSA	18/55 (32.7)
SCC	172/784 (21.9)
S-hCG	3/3 (100)
Pretest diagnosis	n (%)
Head and neck neoplasms	988 (28.4)
Hematopoietic neoplasms	510 (14.6)
Neoplasms of lung, pleura, or mediastinum	507 (14.5)
Hepatobiliary neoplasms	305 (8.8)
Gastrointestinal neoplasms	258 (7.4)
Skin neoplasms	168 (4.8)
Urologic neoplasms	135 (3.9)
Gynecological neoplasms	112 (3.2)
Sarcoidosis	91 (2.6)
Breast neoplasms	67 (1.9)
Brain and spinal neoplasms	65 (1.9)
Others	279 (8.0)

The location of any malignant uptake was determined as A) head and neck, B) chest, C) abdomen, or D) pelvic region. For the classification, the physician was blinded to the CT images and parameters such as maximum standardized uptake value (SUVmax). Diagnostic reports were made based on several factors including SUVmax, the diameter of tumors, visual contrast between the tumors, location of tumors, and changes over time by 2+ physicians each with more than 8 years’ experience in nuclear medicine.

### Image acquisition and reconstruction

All clinical PET/CT studies were performed with either Scanner 1 or Scanner 2. All patients fasted for ≥6 h before the injection of FDG (approx. 4 MBq/kg), and the emission scanning was initiated 60 min post-injection. For Scanner 1, the transaxial and axial fields of view were 68.4 cm and 21.6 cm, respectively. For Scanner 2, the transaxial and axial fields of view were 57.6 cm and 18.0 cm, respectively. Three-min emission scanning in 3D mode was performed for each bed position. Attenuation was corrected with X-CT images acquired without contrast media. Images were reconstructed with an iterative method integrated with (Scanner 1) or without (Scanner 2) a point spread function. For Scanner 2, image reconstruction was reinforced with the time-of-flight algorithm.

Each reconstructed image had a matrix size of 168 × 168 with the voxel size of 4.1 × 4.1 × 2.0 mm for Scanner 1, and a matrix size of 144 × 144 with the voxel size of 4.0 × 4.0 × 4.0 mm for Scanner 2. MIP images (matrix size 168 × 168) were generated by linear interpolation. MIP images were created at increments of 10-degree rotation for up to 180 or 360 degrees. Therefore, 18 or 36 angles of MIP images were generated per patient. In this study, CT images were used only for attenuation correction, not for classification.

### Convolutional neural network (CNN)

A neural network is a computational system that simulates neurons of the brain. Every neural network has input, hidden, and output layers. Each layer has a structure in which multiple nodes are connected by edges. A “deep neural network” is defined as the use of multiple layers for the hidden layer. Machine learning using a deep neural network is called “deep learning.” A convolutional neural network (CNN) is a type of deep neural network that has been proven to be highly efficient in image recognition. CNN does not require predefined image features. We propose the use of a CNN to classify the images of the FDG PET examination.

### Architectures

**Fig. 1:**
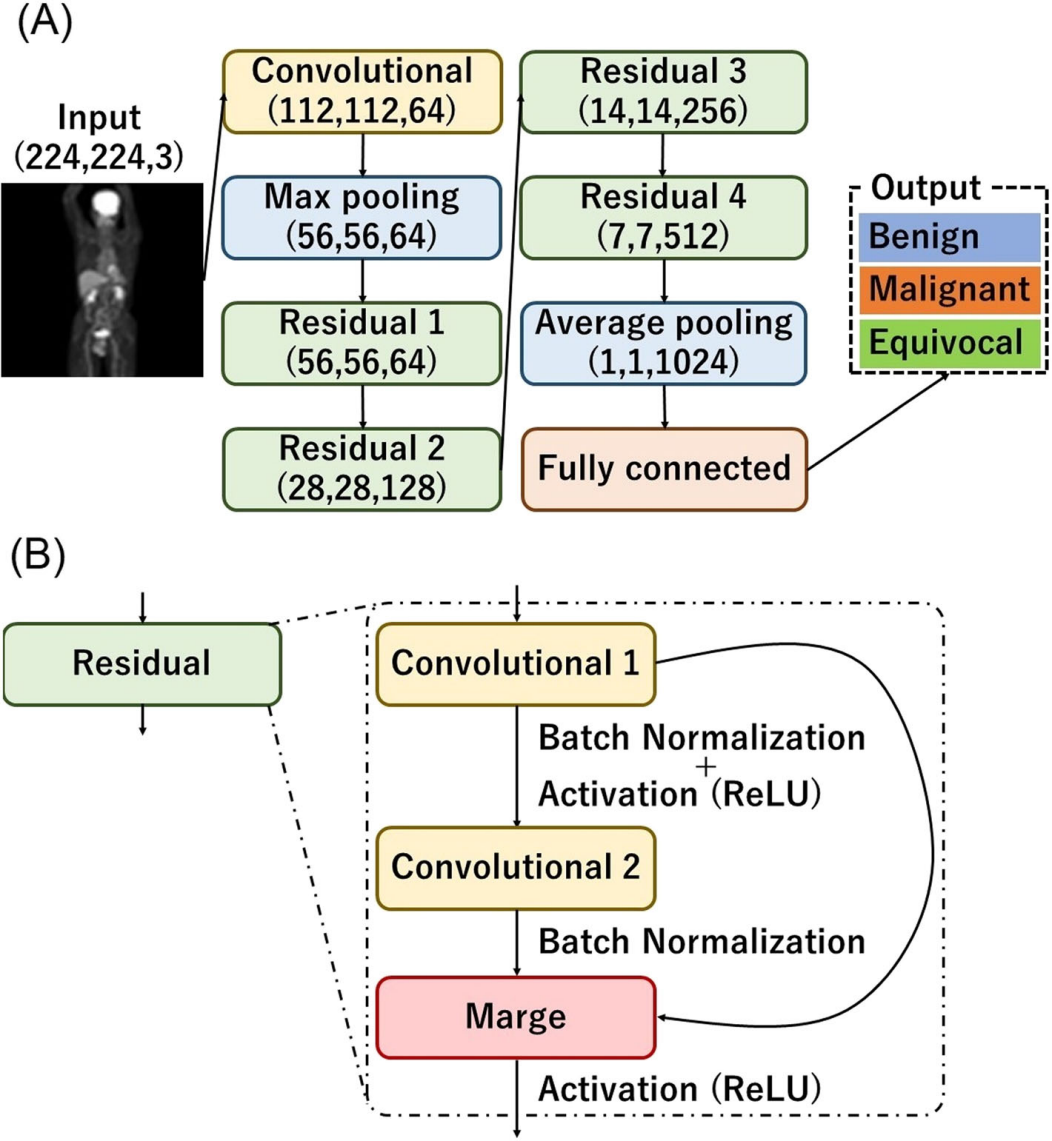
The functional architecture of the CNN. **a** The detailed structure of the CNN used in this study. **b** An internal structure of the residual layer

In this study, we used a network model with the same configuration as ResNet [19]. In the original ResNet, the output layer was classified into 1000 classes. We modified the number of classes to 3. We used this network model to classify whole-body FDG PET images into 1) benign, 2) malignant and 3) equivocal categories. Here we provide details on CNN architectures with the techniques used in this study. The detailed architecture is shown in Fig. 1 and Table 2. Convolution layers create feature-maps that extract image features. Pooling layers have the effect of reducing the amount of data and improving the robustness against misregistration by down-sampling the obtained feature-map. “Residual” is a block that can be said to be a feature of ResNet that combines several layers, thereby solving the conventional gradient disappearance problem. Each neuron in a layer is connected to the corresponding neurons in the previous layer. The architecture of the CNN used in the present study contained five convolutional layers. This network also applied a rectified linear unit (ReLU) function, local response normalization, and softmax layers. The softmax function is defined as follows:

$$ \mathrm{F}\left({x}_i\right)=\frac{\mathit{\exp}\left({x}_i\right)}{\sum \limits_j\mathit{\exp}\left({x}_j\right)} $$

where *x*_*i*_ is the output of the neuron i (i = 1, 2, …, n, with n being the number of neurons belonging to the layer).

**Table 2:** Details of architecture

Layer	Filter Size	Stride	Repeat count	Output Size
Input				(224, 224, 3)
Convolutional	(7, 7)	(2, 2)	1	(112, 112, 64)
Max pooling	(3, 3)	(2, 2)	1	(56, 56, 64)
Residual 1	(3 × 3, 64) (3 × 3, 64)	(1, 1)	3	(56, 56, 64)
Residual 2	(3 × 3, 128) (3 × 3, 128)	(2, 2)	4	(28, 28, 128)
Residual 3	(3 × 3, 256) (3 × 3, 256)	(2, 2)	6	(14, 14, 256)
Residual 4	(3 × 3, 512) (3 × 3, 512)	(2, 2)	3	(7, 7, 512)
Average pooling	(7, 7)	(1, 1)	1	(1, 1, 1024)
Fully connected				(3)

“Residual” contains the following structure. “1. Convolutional layer1, 2. Batch normalization1, 3. Activation layer1 (ReLU), 4. Convolutional layer2, 5. Batch normalization2, 6. Merge layer (Add), 7. Activation layer2 (ReLU)”

### Patient-based classification

The patient-based classification was performed only in the test phase. After test images were classified by CNN, the patient was classified based on the 2 different algorithms (A and B).

Algorithm A:

1. If one or more images of the patient were judged as malignant, the patient was judged as being malignant.
2. If all the images of the patient were judged as benign, the patient was judged as being benign.
3. If none of the above were satisfied, the patient was judged as being equivocal.

Algorithm B:

1. If more than 1/3 of all the images of the patient were judged as malignant, the patient was judged as being malignant.
2. If less than 1/3 of all the images of the patient were judged as malignant and more than 1/3 were judged as equivocal, the patient was judged as being equivocal.
3. If none of the above were satisfied, the patient was judged as being benign.

### Hardware and software environments

This experiment was performed under the following environment:

Operating system, Windows 10 pro 64 bit; CPU, intel Core i7-6700K; GPU, NVIDIA GeForce GTX 1070 8GB; Framework, Keras 2.2.4 and TensorFlow 1.11.0; Language, Python 3.6.7; CNN, the same configuration as ResNet; Optimizer, Adam [20].

## Results

**Fig. 2:**
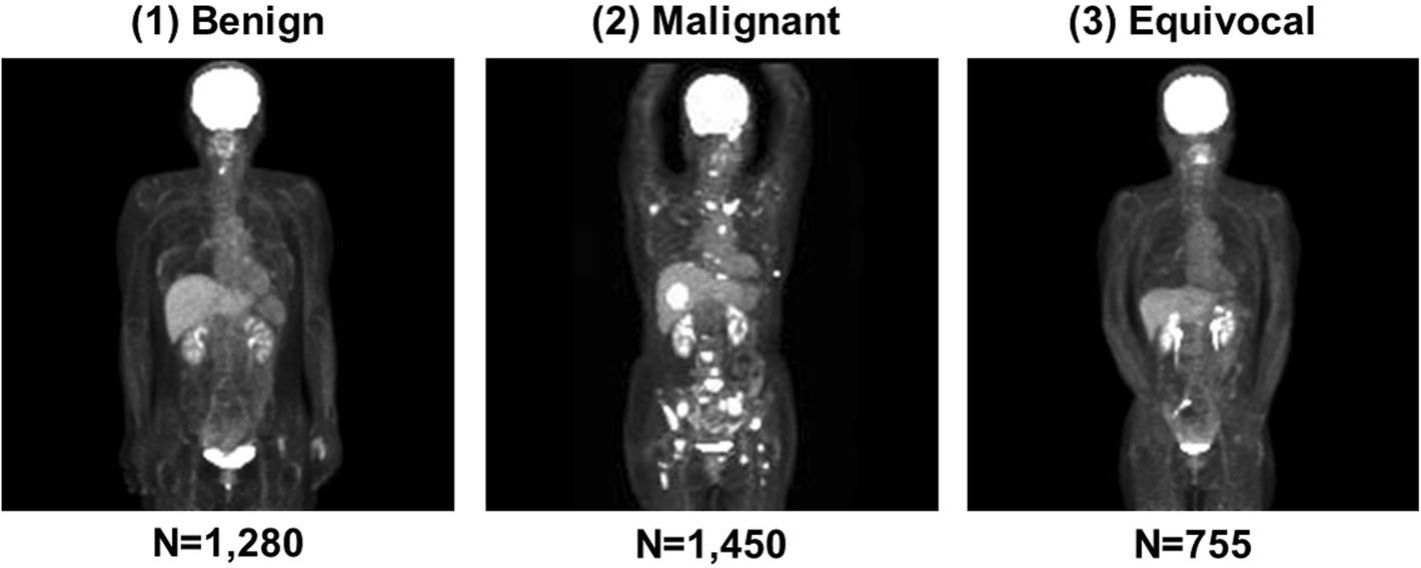
Typical cases in this study. (1) benign patient with physiological uptake in the larynx, (2) malignant uptake patient with multiple metastases to bones and other organs, and (3) equivocal patient with abdominal uptake that was indeterminant between malignant or inflammatory foci

Figure 2 shows typical images of each category. A total of 76,785 maximum intensity projection (MIP) images were investigated. The number of images of benign patients, malignant patients, and equivocal patients was 28,688, 31,751 and 16,346, respectively.

### Experiment 1 (whole-body analysis)

In the image-based prediction, the model was trained for 30 epochs using an early stopping algorithm. The CNN process spent 3.5 h for training and < 0.1 s/ image for prediction. When images of benign patients were given to the learned model, the accuracy was 96.6%. Similarly, the accuracies for images of malignant and equivocal patients were 97.3 and 77.8%, respectively. The results are shown in Table 3 (a). In addition, Table 3 (b) shows the results of recall, compatibility, and F-value calculations.

**Table 3:** Details of Results of Experiments 1 and 2

Experiment 1				
**(a) Image-based**		Correct Label		
		Benign	Malignant	Equivocal
Prediction	Benign	96.6%	2.4%	10.1%
	Malignant	0.3%	97.3%	12.1%
	Equivocal	3.2%	0.2%	77.8%
**(b) Image-based Evaluation Measures**		Recall score	Precision score	F measure
Prediction	Benign	0.966	0.917	0.941
	Malignant	0.973	0.936	0.954
	Equivocal	0.778	0.986	0.87
**(c) Patient-based Algorithm A**		Correct Label		
		Benign	Malignant	Equivocal
Prediction	Benign	91.0%	0.0%	0.0%
	Malignant	9.0%	100.0%	42.5%
	Equivocal	0.0%	0.0%	57.5%
**(d) Patient -based Algorithm A Evaluation Measures**		Recall score	Precision score	F measure
Prediction	Benign	0.910	1.000	0.953
	Malignant	1.000	0.764	0.866
	Equivocal	0.575	1.000	0.730
**(e) Patient-based Algorithm B**		Correct Label		
		Benign	Malignant	Equivocal
Prediction	Benign	99.4%	0.6%	3.8%
	Malignant	0.6%	99.4%	8.8%
	Equivocal	0.0%	0.0%	87.5%
**(f) Patient -based Algorithm B Evaluation Measures**		Recall score	Precision score	F measure
Prediction	Benign	0.994	0.975	0.984
	Malignant	0.994	0.951	0.972
	Equivocal	0.875	1.000	0.933
**Experiment 2**				
**(g) Head and Neck**		Correct Label		
		Benign	Malignant	Equivocal
Prediction	Benign	97.8%	1.7%	3.0%
	Malignant	1.5%	97.3%	0.8%
	Equivocal	0.7%	1.1%	96.2%
**(hd) Chest**		Correct Label		
		Benign	Malignant	Equivocal
Prediction	Benign	98.4%	1.8%	5.9%
	Malignant	0.6%	96.6%	1.6%
	Equivocal	1.0%	1.6%	92.5%
**(i) Abdomen**		Correct Label		
		Benign	Malignant	Equivocal
Prediction	Benign	94.9%	5.7%	7.0%
	Malignant	1.1%	92.8%	2.0%
	Equivocal	4.1%	1.5%	91.0%
**(j) Pelvic region**		Correct Label		
		Benign	Malignant	Equivocal
Prediction	Benign	99.7%	0.4%	2.8%
	Malignant	0.1%	99.6%	1.9%
	Equivocal	0.3%	0.0%	95.3%

**Fig. 3:**
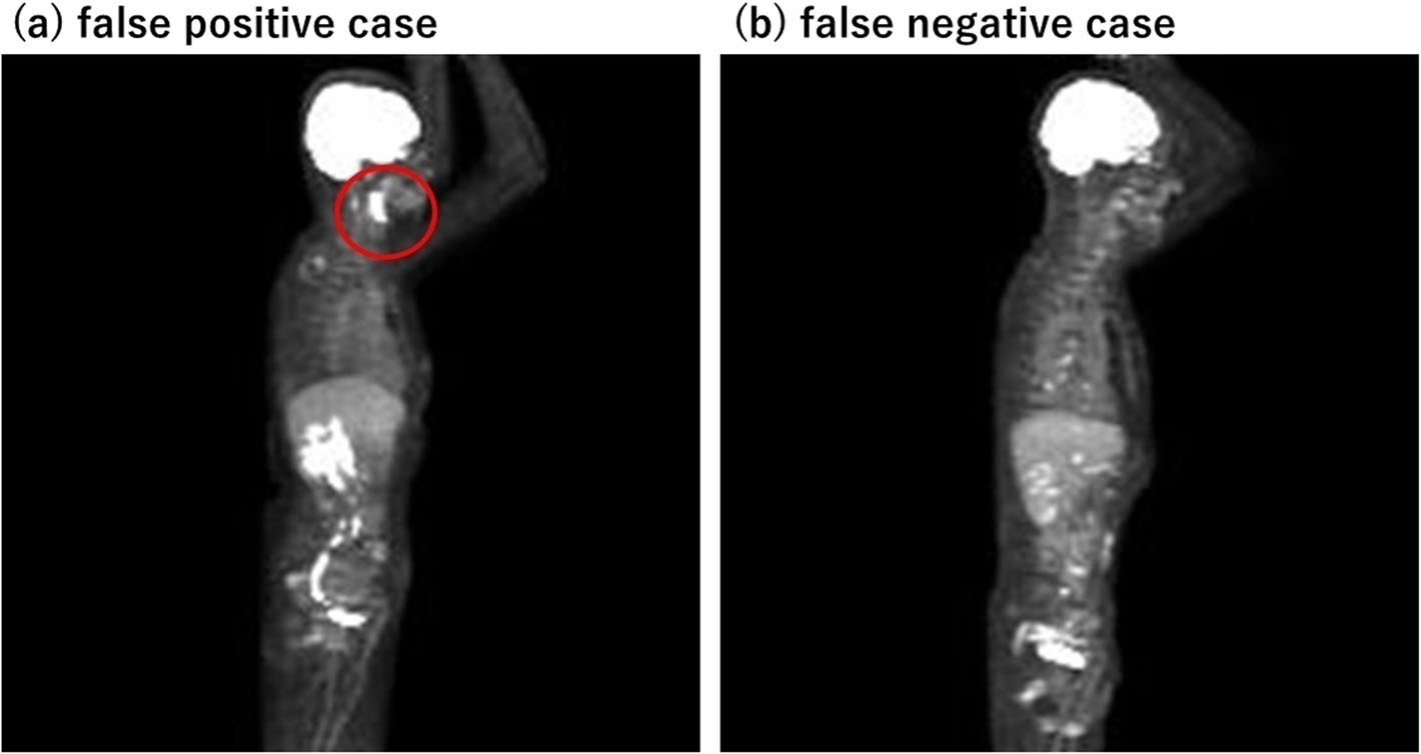
Typical cases whose category was incorrectly classified (**a**, false-positive case; **b**, false-negative case)

In the patient-based classification, we applied algorithms A and B. When the algorithm A was applied, 91.0% of benign patients, 100% of malignant patients, and 57.5% of equivocal patients were correctly predicted. When the algorithm B was applied, 99.4% of benign patients, 99.4% of malignant patients, and 87.5% of equivocal patients were correctly predicted (Table 3c and d). The prediction showed a tendency to fail especially when strong physiological accumulation (e.g., in the larynx) or mild malignant accumulation was present. Typical cases where the neural network failed to predict the proper category are shown in Fig. 3.

### Experiment 2 (region-based analysis)

The same population was used in this experiment as was used in Experiment 1. The model was trained for 33–45 epochs for each dataset using an early stopping algorithm. The CNN process spent 4–5 h for training and < 0.1 s/image for prediction.

In the experiment for the head-and-neck region, a new labeling system was introduced to classify the images into 3 categories: 1) benign in the head-and-neck region, 2) malignant in the head-and-neck region, and 3) equivocal in the head-and-neck region. When images from “malignant in the head-and-neck region” patients were given to the learned model, the accuracy was 97.3%. The accuracy was 97.8 and 96.2% for “benign in the head-and-neck region” patients and “equivocal in the head-and-neck region” patients, respectively.

Similar experiments were performed for the chest, abdominal, and pelvic regions. The details of the results are shown in Table 3 (g)-(j). The accuracy was higher for the pelvic region (95.3–99.7%) than for the abdominal region (91.0–94.9%).

### Experiment 3 (grad-CAM [18])

**Fig. 4:**
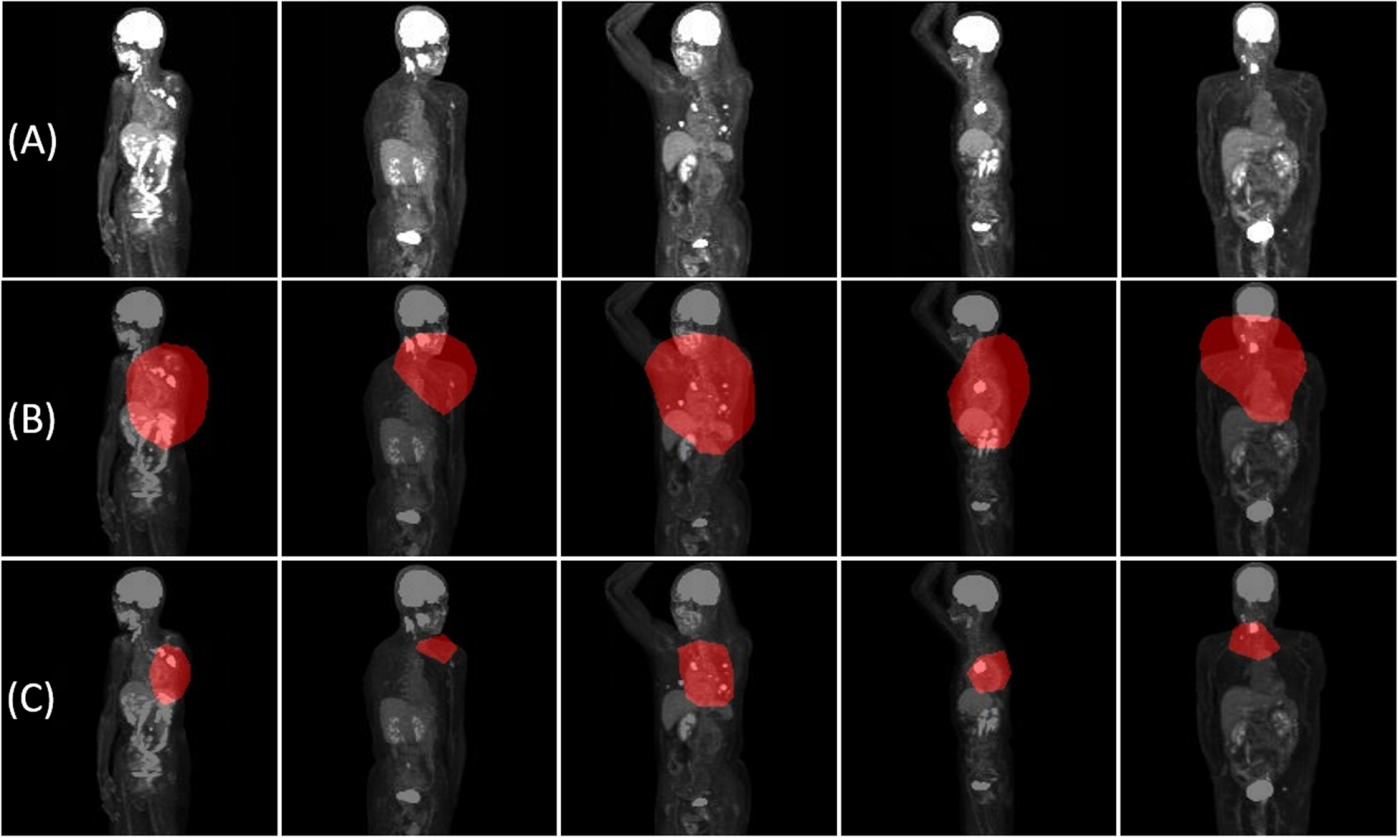
Visualization of classification standard of CNN. **a** Examples of original images input to CNN. **b** Examples of images activated area with the cut-off of 70% maximum by Grad-CAM, highlighting the area of malignant uptake. **c** Examples of images activated area with the cut-off of 90% maximum by Grad-CAM, highlighting the area of malignant uptake

We employed Grad-CAM to identify the part of the image from which the neural network extracted the largest amount of information. Typical examples are shown in Fig. 4. As a result, when the activated area was defined with the cut-off of 70% maximum, 93% of patients had at least one image that showed the activated area covering any part of the tumor. Similarly, when the activated area was defined with the cut-off of 90% maximum, 72% of patients had at least one image that showed the activated area covering any part of the tumor.

## Discussion

In patient-based classification, the neural network predicted correctly both the malignant and benign categories with 99.4% accuracy, although the accuracy for equivocal patients was 87.5%. Therefore, an average probability of 95.4% suggests that CNN may be useful to predict 3-category classification from MIP images of FDG PET. Furthermore, in the prediction of the malignant uptake region, it was classified correctly with probabilities of 97.3% (head-and-neck), 96.6% (chest), 92.8% (abdomen) and 99.6% (pelvic region), respectively. These results suggested that the system may have the potential to help radiologists avoid oversight and misdiagnosis.

To clarify the reasons for the classification failure, we investigated some cases that were incorrectly predicted in Experiment 1. As expected, the most frequent patterns we encountered were strong physiological uptake and weak pathological uptake. In the case shown in Fig. 3a, the physiological accumulation in the oral region was relatively high, which might have caused erroneous prediction. In contrast, another case (Fig. 3b) showed many small lesions with low-to-moderate intensity accumulation, which was erroneously predicted as benign despite the true label being malignant. The equivocal category was more difficult for the neural network to predict; the accuracy was lower than for the other categories. The results may be due to the definition; though common in clinical settings, “equivocal” is a kind of catch-all or “garbage” category for all images not clearly belonging to “malignant” or “benign”; thus, a greater variety of images was included in the equivocal category. We speculate that such a wide range may have made it difficult for the neural network to extract consistent features.

We also conducted patient-based predictions in this study. In patient-based prediction, the accuracy was higher than that in image-based prediction by an ensemble effect. This approach takes advantage of MIP images generated from various angles. More specifically, we applied 2 different algorithms: more sensitive Algorithm A and more specific Algorithm B. The select of algorithm may depend on the purpose of FDG PET/CT.

In general, CNN is said to classify images based on some features of the images. Grad-CAM is a technology that visualizes “the region of AI’s interest”. It could be useful for building explainable AI instead of the black box and thus for gaining the trust of the users. The results of Experiment 3 suggested that, in many cases, CNN responded to the part of the malignant uptake if existed. However, in quantitative assessment, when the cut-off of 70% maximum was used to segment highlight regions, the location of the actual tumor was covered in only 93% cases. There were cases where the AI’s diagnosis was correct although Grad-CAM highlighted non-relevant areas of the images. More studies are needed to clarify whether Grad-CAM or other methods are useful for establishing explainable AI.

The computational complexity becomes enormous when CNN directly learns with 3D images [21–25]. Although we employed MIP images in the current study, an alternative approach may be to provide each slice to CNN. However, even in the case of ‘malignant’ or ‘equivocal’, the tumor is usually localized in some small area and thus most of the slices do not contain abnormal findings. Consequently, a positive vs. negative imbalance problem would disturb efficient learning processes. In this context, MIP seems to be advantageous for a CNN as most MIP images of malignant patients contain accumulation in the image somewhere unless a stronger physiological accumulation (e.g., brain or bladder) hides the malignant uptake. In contrast, in 2D axial images or 3D images, tumor uptake is not hidden by physiological uptake. Therefore, we speculate that the prediction accuracy could be improved by using 2D axial images or 3D images if an appropriate neural network architecture is used.

In this study, we used only 2 scanners, but further studies are needed to reveal what will happen when more scanners are investigated. For instance, what if the numbers of examinations from various scanners are imbalanced? What if a particular disease is imaged by some scanners but not by the other scanners? There is a possibility that the AI system cannot make a correct evaluation in such cases. The AI system should be tested using “real-world data” before using it in clinical settings.

Some approaches could further improve the accuracy. In this research, in order to reduce the learning cost, we used a network that is equivalent to ResNet-50 [19], which is a relatively simple version of the “ResNet” family. In fact, ResNet systems with deeper layers can be built technically. More recently, various networks based on ResNet have been developed and demonstrated to have high performance [26, 27]. From the viewpoint of big-data science, it is also important to increase the number of images for further improvement in diagnostic accuracy.

There are many other AI algorithms that can be used for PET image classification and detection. In a recent study by Zhao et al., they used the so-called 2.5D U-Net to detect lesions on ^68^Ga-PSMA-11 PET-CT images for prostate cancer [28]. They trained the CNN using not fully 3D images but axial, coronal, and sagittal images in order to simulate the workflow of physicians and save computational and memory resources. They reported that the network achieved 99% precision, 99% recall, and 99% F1 score. Not only U-Net [29] as an image segmentation method but also regional CNN (RCNN) and M2Det [30] as object extraction methods, may be useful to localize the lesion. In a study by Yan K et al., MR image segmentation was performed using a deep learning-based technology named the Propagation Deep Neural Network (P-DNN). It has been reported that by using P-DNN, the prostate was successfully extracted from MR images with a similarity of 84.13 ± 5.18% (dice similarity coefficient) [31]. On the other hand, these methods also have a problem that enormous time is required to create training data.

The oversight rate (i.e., the rate of misclassifying malignant images as benign ones) was 0.6%. We think that the rate is small but not satisfactory. As we consider the current system will contribute to radiologists as a double-checking system, decreasing oversight is much more important to decreasing the false-positive rate. We are planning experiments to decrease the oversight rate by adding the CT data to CNN.

This study has some limitations. First, this model can only deal with FDG PET MIP images in the imaging range from the head to the knees; correct prediction is much more difficult when spot images or whole-body images from the head to the toes are given. Future studies will use RCNN to solve the problem. Second, less FDG-avid lesions such as pancreatic cancer cannot be classified only with MIP images, and there is a possibility that it cannot be labeled correctly. Third, we applied patient-based labeling but not image-based labeling. Thus, some MIP images of particular angles may be labeled as ‘malignant’ but do not visualize the tumor that is hidden by physiological uptake. To improve the quality of training data, each image within the patient should be labeled separately although it takes plenty of time. Finally, the cases were classified by a nuclear medicine physician but were not based on a pathological diagnosis.

## Conclusion

The CNN-based system successfully classified whole-body FDG PET images into 3 categories in whole-body and region-based analyses. These data suggested that MIP images were useful for classifying PET images and that the AI could be helpful for physicians as a double-checking system to prevent oversight and misdiagnosis. Before using AI in clinical settings, more advanced CNN architectures and prospective studies are needed to improve and validate the results.

## Data Availability

The datasets used and/or analyzed during the current study are available from the corresponding author on reasonable request.

## Abbreviations

AI: Artificial intelligence
CNN: Convolutional neural network
CT: Computed tomography
FDG: ^18^F-fluorodeoxyglucose
Grad-CAM: Gradient-weighted Class Activation Mapping
MIP: Maximum intensity projection
PET: Positron emission tomography
RCNN: Regional convolutional neural network
ReLU: Rectified linear unit
ResNet: Residual network
SUVmax: Maximum standardized uptake value
